# Complete Genome Sequence of a Canadian Strain of Escherichia coli with Multiple Metal and Antimicrobial Resistance Genes That Was Isolated from Municipal Biosolids

**DOI:** 10.1128/mra.00083-23

**Published:** 2023-04-17

**Authors:** Mingsong Kang, Philippe Charron, Emily Hoover, Jiewen Guan, Isaac Firth, Sohail Naushad, Hongsheng Huang

**Affiliations:** a Canadian Food Inspection Agency, Ottawa Laboratory-Fallowfield, Ottawa, Ontario, Canada; University of Maryland School of Medicine

## Abstract

This announcement reports the complete genome sequence of a non-Shiga toxin-producing Escherichia coli strain that was isolated from municipal biosolids collected from a Canadian wastewater treatment plant. This strain contains multiple metal, antimicrobial, and heat resistance genes, as determined by genome sequencing, and could be a useful bacterial model for future studies.

## ANNOUNCEMENT

Escherichia coli is a Gram-negative bacterium, either harmless or pathogenic, that is commonly found in the intestines of humans and animals and in environmental habitats, including agricultural soils and biosolids (1–3). This article reports the genome sequence of an E. coli strain (HH107) that was isolated from a posttreated (biosolids) sample collected from a Canadian wastewater treatment plant in 2010. The strain was isolated by enrichment in lauryl sulfate tryptose broth at 35°C for 24 h, followed by E. coli broth at 45°C for 24 h, and isolation using Levine's eosin methylene blue agar at 35°C for 24 h (4). The strain was identified as E. coli using API 20 E (bioMérieux Canada Inc.) and confirmed by genome sequencing.

Genomic DNA of HH107 was extracted from an overnight culture that had been grown from a single colony in tryptic soy broth using the NanoBind CBB kit (Pacific Biosciences [PacBio], USA), with subsequent treatment using the Short Read Eliminator XS kit (PacBio). Illumina sequencing was conducted by library preparation using a DNA preparation kit (Illumina, USA) and sequencing on a MiSeq platform (Illumina) using the MiSeq reagent kit v3 to generate a total of 2,684,745 paired-end (300-bp) reads, which were filtered and trimmed using Fastp v0.23.2 (5). Nanopore sequencing was performed by MinION library preparation using a ligation sequencing-native barcoding kit (SQK-NBD112.24; Oxford Nanopore Technologies, UK) without shearing and sequencing using a FLO-MIN112 (R10.4.1) flow cell on a MinION Mk1B device. A total of 40,436 reads (*N*_50_ of 17,401 bp) were obtained, followed by base calling using Guppy v6.1.2, trimming using Porechop v0.2.4, and filtering using NanoFilt v2.8.0 (6). Illumina and MinION reads were hybrid assembled using Unicycler v0.5.0 (7). The circularity of the genome and genome rotation using *dnaA* as the starting point were determined by Unicycler v0.5.0. The coverage depth (262×) was assessed using SAMtools v1.13 (8). Gene predictions and annotations were performed using NCBI Prokaryotic Genome Annotation Pipeline (PGAP) v6.4 (9). Metal resistance, heat resistance, and antimicrobial resistance (AMR) genes were identified using AMRFinderPlus v3.11.2 with database v2022-12-19.1 (10). The plasmids were identified by PlasmidFinder v2.0.1 using database v2023-01-12 (11), and prophage sequences were analyzed using the PHASTER web server (12). The serotype was identified using SeroTypeFinder v2.0.1 (13), and pathogenicity was predicted using PathogenFinder v1.1 (14) and VirulenceFinder v2.0.3 (15). Default parameters were used for all of the bioinformatic tools.

The isolate HH107 was predicted to be non-Shiga toxin-producing E. coli O18ab:H14. Its genome contains a single chromosome and three plasmids. Table 1 presents detailed information on total length, chromosome size, GC content, protein count, prophage count, AMR, heavy metal resistance, and heat resistance genes, and virulence genes. The median total length, number of coding sequences (CDSs), and GC content of E. coli genome assemblies in GenBank are similar to those of this E. coli strain (Table 1).

**TABLE 1:** Genomic characteristics of the E. coli strain isolated from a biosolids sample

Strain	No. of contigs	No. of plasmids	Total length (Mb)	Chromosome size (bp)	GC content (%)	No. of proteins	Genes related to AMR^*a*^	Genes related to metal resistance^*a*^	Genes related to heat resistance^*a*^	Virulence genes^*b*^	No. of intact prophages
HH107	4	3	5.05	4,795,218	50.9	4,632	*acrF*, *mdtM*, *blaEC*, *glpT* (4 genes)	*silE*, *silS*, *silR*, *silC*, *silF*, *silB*, *silA*, *silP*, *pcoA*, *pcoB*, *pcoC*, *pcoD*, *pcoR*, *pcoS*, *pcoE*, *arsR*, *arsD*, *arsA*, *arsC* (19 genes)	*hsp20*, *clpK*, *shsP*, *yfdX1*, *yfdX2*, *hdeD-GI*, *trxLHR*, *kefB-GI*, *psi-GI* (9 genes)	*anr*, *clpK1*, *csgA*, *fdeC*, *fimH*, *gad*, *hha*, *hlyA*, *hlyE*, *nlpI*, *terC*, *traJ*, *traT*, *yehA*, *yehB*, *yehC*, *yehD* (17 genes)	4
Median GenBank sequences^*c*^			5.10		50.6	4,724					

a AMR, heavy metal resistance, and heat resistance genes were predicted by AMRFinderPlus.

b Virulence genes were predicted by VirulenceFinder.

c Data summarized on 27 February 2023 using 33,383 genome assemblies are available in a genome assembly and annotation report (https://www.ncbi.nlm.nih.gov/genome/browse#!/prokaryotes/167).

### Data availability.

The chromosome and plasmid sequences of strain HH107 have been deposited in GenBank under accession numbers CP116405, CP116406, CP116407, and CP116408. MinION and MiSeq raw data are available in the NCBI Sequence Read Archive (SRA) under SRA accession numbers SRR23100672 and SRR23100675, respectively.
